# Factors related to leader implementation of a nationally disseminated community-based exercise program: a cross-sectional study

**DOI:** 10.1186/1479-5868-5-62

**Published:** 2008-12-04

**Authors:** Rebecca A Seguin, Ruth Palombo, Christina D Economos, Raymond Hyatt, Julia Kuder, Miriam E Nelson

**Affiliations:** 1 John Hancock Center for Physical Activity and Nutrition, Tufts University, Boston, MA, USA; 2 Friedman School of Nutrition Science and Policy, Tufts University, Boston, MA, USA; 3 Jonathan M. Tisch College of Citizenship and Public Service, Tufts University, Medford, MA, USA; 4 Department of Public Health and Family Medicine, School of Medicine, Tufts University; Boston, MA, USA

## Abstract

**Background:**

The benefits of community-based health programs are widely recognized. However, research examining factors related to community leaders' characteristics and roles in implementation is limited.

**Methods:**

The purpose of this cross-sectional study was to use a social ecological framework of variables to explore and describe the relationships between socioeconomic, personal/behavioral, programmatic, leadership, and community-level social and demographic characteristics as they relate to the implementation of an evidence-based strength training program by community leaders. Eight-hundred fifty-four trained program leaders in 43 states were invited to participate in either an online or mail survey. Corresponding community-level characteristics were also collected. Programmatic details were obtained from those who implemented. Four-hundred eighty-seven program leaders responded to the survey (response rate = 57%), 78% online and 22% by mail.

**Results:**

Of the 487 respondents, 270 implemented the program (55%). One or more factors from each category – professional, socioeconomic, personal/behavioral, and leadership characteristics – were significantly different between implementers and non-implementers, determined by chi square or student's *t*-tests as appropriate. Implementers reported higher levels of strength training participation, current and lifetime physical activity, perceived support, and leadership competence (all p < 0.05). Logistic regression analysis revealed a positive association between implementation and fitness credentials/certification (p = 0.003), program-specific self-efficacy (p = 0.002), and support-focused leadership (p = 0.006), and a negative association between implementation and educational attainment (p = 0.002).

**Conclusion:**

Among this sample of trained leaders, several factors within the professional, socioeconomic, personal/behavioral, and leadership categories were related to whether they implemented a community-based exercise program. It may benefit future community-based physical activity program disseminations to consider these factors when selecting and training leaders.

## Background

An essential component of community-based physical activity programs are the leaders who implement them [1-4]. Community program leaders serve many roles in implementation – from soliciting participation and teaching classes to providing motivation, inspiration, and feedback to participants [3,5,6]. Leaders may also carry out a range of administrative and logistical tasks.

Leaders often possess a wide variety of backgrounds and skills, which inevitably influence the programs they implement and the participants who receive them. In community-based public health program evaluations, there are varying types and degrees of success (related to implementation, participation, biologic/health-related outcomes, and beyond); it is likely that the characteristics of the leaders and communities contribute to this variability – independently and collectively [7-12].

Currently, there is a gap within the public health literature by which a comprehensive model could be developed and utilized to identify, select, and strategically train community-based leaders to maximize their skills and capacity. General literature on leadership, however, lends insight to factors and characteristics worthy of exploration. For example, it has been proposed that leadership is influenced by the leader's personal characteristics. Research suggests that such characteristics can be defined on a variety of levels, such as age, education level, or in a capacity that is more descriptive of life experiences and personal habits, such as eating habits or exercise practices. In this context, a study may seek to determine which factor is important to program implementation: formal training or prior program implementation experience? Perhaps neither; perhaps both. Also, does it matter whether the leaders themselves model the behavior they are trying to encourage? Personal commitment and/or experience may affect the execution of implementation [5,13-20].

Other theories on leadership suggest that while personal characteristics are important, situational factors such as support, the organization, or environment may interact with individual factors and modify effectiveness accordingly [7,13,15,17,21-23]. These theories imply that even if all essential components are in place at the individual (leader) level, the context or situational characteristics play an inevitably crucial role. An environment where appropriate resources are allocated toward development and where learning is rewarded is also essential to the development of an effective leader. This concept may be thought of as quality of institutional or organizational leadership [14,20,23-26].

What is commonly agreed upon in this area is that appropriate leadership is important to bring about change within a group, and that greater levels of change require leadership that is more dynamic. It has been suggested that the most effective leaders are those who rely on not one but multiple leadership styles and those who are able to adapt depending on the situation [23,26].

Considered succinctly, optimizing leadership is likely a function of both personal and environmental factors that facilitate program implementation and sustainability through separate and collective action [7,13,15,17,21]. Hence, the characteristics of the target group, nature of the work, type of group structure, and nature of the external environment may all influence what could be considered "successful" leadership [13,17,19,27]. Therefore, examining and understanding the effects of leadership, individual, and situational characteristics may confer enhanced success in implementing and sustaining a variety of community programs [3,15,18,24,28-31].

There is emerging popularity and demand for evidence-based health promotion programs. Despite the recognized importance of community program leadership and an extensive body of literature related to the leadership for the business sector [14,16,22,32], research in this area as it relates to public health is limited, in both quantity and depth. The multilevel influence embedded within community-based programs implementation and dissemination efforts invite the social ecological model as a viable and practical framework for examination [33,34]. The findings presented here aim to expand understanding of the role of leadership in community-based program implementation, and to generate hypotheses for future studies.

### Study objective

**Figure 1:**
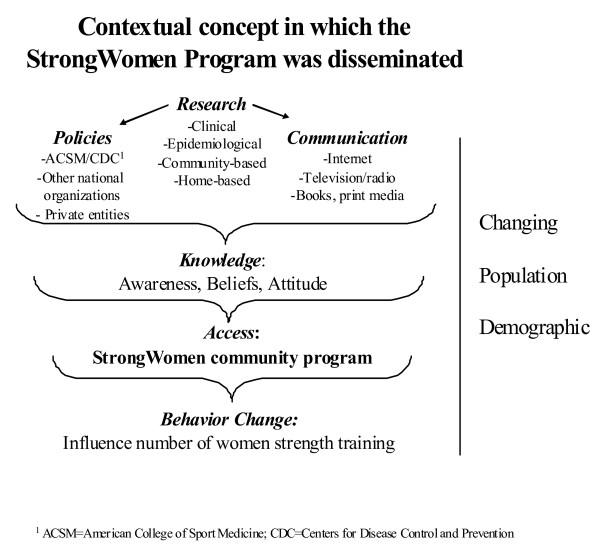
**Dissemination Context**. Context for development and dissemination of the StrongWomen Program, a community-based strength training program targeted to women aged 40 and older. This figure was previously published [6].

**Figure 2:**
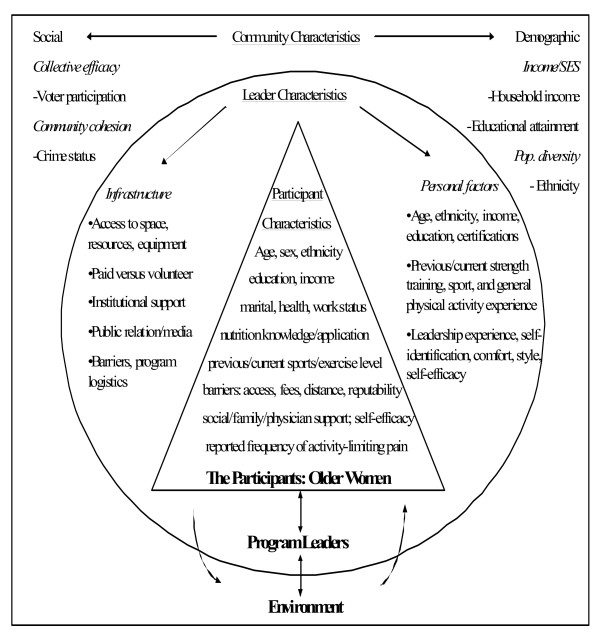
**Social Ecological Framework**. Social ecological framework describing the leader, participant, and community characteristics examined in this study, and how they may be related to implementation (leaders) and adherence (participants). The community-level characteristics also help to describe the larger contextual landscape of the dissemination environment.

The purpose of this study was to explore and describe the relationships between socioeconomic, personal/behavioral, programmatic, leadership, and community-level social and demographic characteristics as they relate to the implementation of an evidence-based strength training program by community leaders. This was a cross-sectional design that utilized a convenience sample of leaders from the StrongWomen Program (SWP) – a nationally disseminated community strength training program targeted to midlife and older women [6]. The primary hypothesis stated that implementation of the SWP would be positively associated with a community leader's previous strength training experience, support, and leadership characteristics compared to leaders who did not implement the program. The contextual framework for the dissemination of the SWP is shown in Figure 1; an extensive review of the national dissemination of the SWP – including the training workshop, curriculum, and programmatic details – have been previously published [6]. The social-ecological framework of variables for this research is shown in Figure 2.

## Methods

The SWP was designed as a community-based program to be implemented as twice weekly group strength training classes lasting eight to twelve weeks in community settings. At the time of survey administration (June 2006), thirty-nine day-long workshops had been conducted from May 2003–June 2006 – training 854 community leaders from forty-three states to lead the SWP. Program leaders are most commonly from nonprofit organizations such as the USDA Cooperative State Research, Extension, and Education Service, hospital-based wellness centers, and community/recreation centers [6]. Contact information for trained leaders is tracked using a detailed database that includes phone, email, address, and other professional information.

### Survey design and development

The preliminary survey development involved the synthesis of findings from three sources: approximately 500 post-workshop evaluations completed by leaders; data from a pilot phone survey conducted in September 2004; and group program participant interviews previously conducted during program site visits from 2004–2005 [6,35]. Those data were used to compile a working draft concept and content table, which framed the survey outline. Phone interviews were then conducted with five program leaders and five program administrators from a range of geographic locations to expand upon and revise the outline. Drafts of the survey were reviewed and pilot tested internally among the research team and selected colleagues, in both an Internet-based and paper-based format.

Following the first round of modifications, the survey was then pilot tested in the online and paper formats with ten program leaders from six states, including the five leaders who initially participated in the phone interviews; all were subsequently excluded from the final survey participation. Following revisions, the survey along with all related materials (i.e. cover letter/cover email inviting leaders to participate, consent form, etc.) were approved by the Tufts University Human Investigation Review Board (IRB approval #7049).

### Survey data collection

Eight-hundred fifty-four leaders were invited to participate in the survey beginning in June 2006. All leaders who provided email addresses at workshop trainings in which they participated received the email invitation, which included a link to the online consent and survey. Those who did not provide an email address as well as those who responded asking for a printed version, were mailed the paper-based version, which included a written consent form. After the initial email and paper mailing, all nonrespondents received both the email-based and paper-based invitations on two subsequent release dates – each separated by approximately three weeks (for a total of three survey invitations). All respondents were required to give informed consent to participate. Following survey submission, all respondents were mailed a thank you letter.

All survey data were collected over a three-month period. Paper survey data were entered into SPSS Data Builder 14.0; Internet-based survey data were downloaded to a Microsoft Excel spreadsheet and subsequently converted to the SPSS 14.0 format. Data from the paper surveys and online survey were merged. Data cleaning and recoding as well as all data analysis were conducted using SPSS 14.0 [36].

Of the 854 leaders surveyed, 487 completed the survey, yielding a 57% response rate. Of the 487 survey respondents, 381 were online respondents (78%) and 106 were mail respondents (22%). Analyses were conducted between online and paper-based respondents in terms of personal characteristics (i.e. age, sex, race, education, income, etc.) as well as program-related characteristic (i.e. implementation rates, participant compliance, etc.). Using chi square to compare categorical variables and *t *tests to compare continuous variables, no statistically significant differences were found between the online and mail respondents; therefore, all data were analyzed and are shown together.

### Outcome measurements

#### Implementation

Survey respondents (herein "respondents") were asked (no/yes answer format) if they had implemented at least one program (twice weekly SWP classes) following their workshop attendance. Individuals who answered *yes *were classified as *implementers*. This was the primary outcome of interest, and the dependent variable (0 = no, 1 = yes) for the logistic regression analysis.

#### Socioeconomic and professional factors

Socioeconomic characteristics included the following: age, sex, race, marital status, educational attainment (i.e. bachelors level), income, and work status; questions were adapted from the U.S. Census Bureau *American Community Survey *and the *Behavioral Risk Factor Surveillance System Survey Questionnaire *[37,38]. Professional variables collected included educational degree concentration (i.e. physical therapy); fitness certification/credential attainment; professional title and job responsibilities (if applicable); and employer name and type of organization (if applicable).

#### Program-related personal factors

The program-related personal characteristics of respondents asked leaders to categorize their current and previous activity level, current and previous sports participation, past and current strength training participation, change in activity since workshop attendance, and the activity level of the leader's significant other (if applicable). Physical activity and nutrition topic areas were derived from the National Health Interview Survey; specific questions were developed, pilot tested, and administered for this survey [38].

#### Leadership factors

Respondents answered questions about their program-specific self-efficacy related to their confidence to overcome potential challenges related to social, physical, and logistical aspects of program implementation; a combined overall score for program-related self-efficacy was calculated. The self-efficacy questions were adapted from the General Self-Efficacy Scale [39]. To characterize leadership competence, respondents were asked whether they self-identify as a leader and whether they are comfortable leading people in activities; responses were yes/no [27]. To characterize leadership style, four primary categories from well-established leadership inventories were utilized: organization, support, communication, and conflict resolution [27,40]. Respondents answered eight questions that were used to characterize their leadership style, within the aforementioned four categories. The leadership competence and leadership characteristics questions were informed by the Leadership Practices Inventory and other relevant sources, and developed for this survey [27,40]. Respondents also reported perceived support from friends, family, and/or their supervisor to implement the program as well as the reason they attended the training workshop [41].

#### Demographic comparisons

To assess socioeconomic status (SES), survey respondents indicated their educational attainment, household income level, and race (individual level). These variables were also collected at the community level (respondent zip codes) and at the national level using 2004 U.S. Census data [37]. Means were compared for education, income, and race between the individual level and community level, between the individual and national levels, and between the community and national levels. In addition, voter participation rates and crime rates were collected at the community level and national level, as indicators for community participation and collective efficacy, respectively. Statistical means for each of the variables were compared at the community and the national level [42,43].

### Statistical analyses

The chi-square test was used to compare implementers to non-implementers on categorical variables, and the *t *test was used to compare continuous variables. Logistic regressions examined factors related to implementation. The *a priori *hypothesized model was specified as: *implementation = educational attainment + income + program-related self-efficacy + fitness certification/credential + support-focused leadership style + previous strength training experience + age*. Additional model details are provided in the *Results *section of this manuscript. The data were analyzed using SPSS 14.0 [36]. In addition, five community-level variables were examined that describe specific aspects of community: education and income (SES); race (population diversity); crime rates (social cohesion), and voter participation (collective efficacy) [44].

## Results

### Implementation

**Figure 3:**
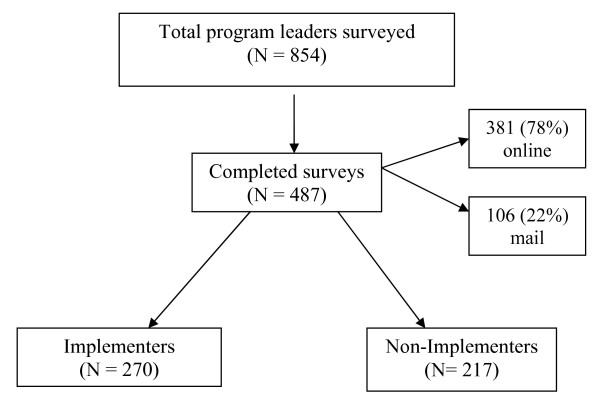
**Program Leader Survey Response**. Graphic of overall survey response rate, percentages of paper and online surveys received, and breakdown of program implementers and non-implementers.

Of the 487 respondents, 270 (55%) were classified as implementers; 217 (45%) were classified as non-implementers. See Figure 3.

### Socioeconomic and professional factors

The majority of respondents were educated white (93%), female (98%), and working full- or part-time (≥ 90% collectively). Age, sex, race, marital status, household income, and work status were not different between non-implementers and implementers. Non-implementers reported significantly greater masters-level educational attainment compared to implementers (p = 0.003); implementers reported greater bachelor-level attainment compared to non-implementers (p = 0.034). Data presented in Table 1. In addition, the occupation distribution of program leaders is presented in Table 2.

**Table 1:** Socioeconomic and Professional Characteristics.

	*Non-Implementers* *N = 217*^*a*^	*Implementers* *N = 270*^*b*^	*P-Value*
Age in years, mean (SD)	50 (10)	50 (11)	0.965

Sex, % female	98	98	0.986

Race, % white	93	93	0.953

Married/living with domestic partner, %	73	74	0.465

Education level, %			
- Some HS	-	-	1.000
- HS grad	3	3	1.000
- Some college	10	15	0.174
- BS	29	38	0.034
- MS+	58	44	0.003

Household income, %			
- <20K	5	3	0.799
- 20-49999	18	26	0.059
- 50-74999	33	30	0.548
- 75-100K	26	21	0.209
- >100K	20	20	0.845

Work status, %			
- Full time	69	70	0.862
- Part time	21	21	0.963
- Volunteer only	6	5	0.698
- No work	4	4	1.000

Fitness credential/certification, %	24	35	0.005

Due to the nature of survey data, sample size varies by question.

^a ^– sample size range: n = 189–217

^b ^– sample size range: n = 236–270

**Table 2:** Occupation distribution of program leaders^a^

**Jobs**	**Percent**
Extension Agent	43.0%

Fitness Instructor/Personal Trainer	7.8%

Physician/Nurse	4.1%

Physical Therapist	1.8%

Nutritionist/Dietician	1.6%

Other Healthcare	5.9%

Community Educator/Community Organizer	5.8%

Academic Educator	2.0%

Student	1.2%

Self-employed	1.4%

Other	10.9%

Due to the nature of survey data, sample size varies by question.

^a ^– sample size: n = 754

### Program-related personal factors

Implementers reported significantly lower current physical inactivity levels, greater current strength training habits, and increased post-workshop physical activity levels compared to non-implementers (all p < 0.001). Lifetime physical activity level, current and prior sports participation, spouse/domestic partner activity level, and prior strength training experiences were not different between non-implementers and implementers. Data presented in Table 3.

**Table 3:** Program-Related Personal/Behavioral Characteristics

	*Non-Implementers* *N = 217*^a^	*Implementers* *N = 270*^b^	*P-Value*
Current PA level, %			
- Not active	10	2	<0.001
- Somewhat active	42	40	0.595
- Active	48	58	0.031

Lifetime PA level, %			
- Not active	4	4	1.000
- Somewhat active	54	46	0.113
- Active	42	50	0.136

Current sports participation, %	17	23	0.124

Prior sports participation, %	58	65	0.125

Significant other is active, %^c^	75	80	0.294

Prior strength training experience, %	42	47	0.263

Currently strength training regularly, % (at least 1–2 times per week)	59	85	<0.001

Change in activity level since workshop, %			
- Less active	6	-	0.001
- About the same	69	54	0.002
- More active	25	46	<0.001

Due to the nature of survey data, sample size varies by question.

^a ^– sample size range: n = 212–216

^b ^– sample size range: n = 260–270

^c ^– sample size for non-implementers, n = 157; implementers, n = 183

### Leadership characteristics

Implementers reported significantly greater perceived support from their friends, families, and supervisors compared to non-implementers (p < 0.001) as well as greater levels of program-related self-efficacy (p = 0.003) and leadership competence (greater comfort leading others, p = 0.007 and stronger leader self-identification, p = 0.049) compared to non-implementers. Reasons for workshop attendance also differed significantly between the groups. A significantly greater percentage of non-implementers reported "personal health reasons" as their primary reason for attendance (p = 0.002), whereas implementers reported "to implement a program" and "supervisors suggestion" as their reasons compared to non-implementers, although the differences were not significant (p = 0.097 and p = 0.074, respectively). Data presented in Table 4.

**Table 4:** Leadership Characteristics: Support, Self-Identification, Comfort, and Self-Efficacy

	*Non-Implementers* *N = 217*	*Implementers* *N = 270*	*P-Value*
Friends/family/supervisor support of program involvement, %^a^	93	100	<0.001

Reason for workshop attendance, %^b^			
- Supervisor's suggestion	9	14	0.074
- Personal health reasons	32	19	0.002
- To implement a program	41	49	0.097
- Other	18	18	0.741

Program related self-efficacy, mean (SD)^c^	2.76 (0.60)	2.93 (0.29)	0.003

Self-identify as a "leader", %^b^	86	94	0.049

Comfortable leading friends or strangers in an activity, %^b^	95	99	0.007

Due to the nature of survey data, sample size varies by question. Range is noted below.

^a ^– sample size for non-implementers, n = 146; implementers, n = 238;

^b ^– sample size range for non-implementers, n = 202–215; implementers, n = 250–265

^c ^– sample size for non-implementers, n = 168; implementers, n = 229

### Program logistics and barriers

Implementers reported details regarding the SWPs they implemented. Mean months ± SD between workshop attendance and program implementation was 5.1 ± 5.5. Other details included duration of program session, the number of days per week that classes meet, length of class sessions, number of participants per class, peer leader program help, participant attendance rate. They also reported their reasons for implementing, sustaining, and no longer leading programs, as applicable, as well as compensation related to leading the program. Those data are presented in Table 5.

**Table 5:** Program Characteristics (Implementers Only)^a^

	**Mean (SD)**
Duration of program session, weeks	10 (2.5)

Days per week classes meet	2 (0.6)

Length of class sessions, minutes	57 (12)

Number of participants per class	13 (11)

	**Percent**

Peer leader helps with programs, %	72

Attendance rate of participants, %	84

Reason for implementation, %	
- To help others	38
- New professional goal	19
- Supervisor's suggestion	12
- Community involvement	11
- Other	20

Reason for continuing to run the program, %^b^	
- To help others	38
- I enjoy strength training	13
- Community involvement	13
- New professional goal	7
- Supervisor request	6
- Other	23

Reason for no longer leading the program, %^c^	
- A volunteer/colleague took over	27
- Due to time/scheduling conflicts	20
- Not enough participant interest	10
- Job no longer supportive	7
- Other	36

Compensation for running the program, %	
- It's part of my job/salary	51
- It's 100% volunteer (no pay)	38
- Other	11

Due to the nature of survey data, sample size varies by question. Range is noted below.

^a ^– sample size range: n = 246–270 (unless noted otherwise below)

^b ^– sample size: n = 206;

^c ^– sample size: n = 103

Reported barriers to implementation were different between implementers and non-implementers. Compared to non-implementers, implementers reported finding participants as a barrier to starting a program (p < 0.001). Compared to implementers, non-implementers reported being too busy and not having enough support as their barriers (p = 0.01 and p < 0.001, respectively). Data not shown.

### Demographic comparison

Individual, community, and national-level comparisons for education, income, and race as well as community and national-level voter participation and crime rates for leader communities are shown in Tables 6. At the individual-level, respondents had higher levels of education, higher household income, and less racial diversity than their respective communities (all p < 0.001), and their respective communities had higher levels of education, higher household income, less racial diversity compared to the national levels (all p < 0.001). In addition, respondents' respective communities had higher voter participation rates and higher crime rates compared to the country overall (p < 0.001 and p = 0.025, respectively).

**Table 6:** Leader Communities: Individual, Community-level, and National Demographic Comparisons^a,b^

	*Individual level* *(all trained leaders)*	*Community-level* *(leaders' reported zip code)*	*National-level* *(zip code data, 2004 US Census)*
	Mean (SD)	Mean (SD)	Mean (SD)

Education level^c^	4.3 (0.84)	2.68 (0.42)	2.48 (0.44)

Household income^d^	3.33 (1.14)	2.27 (0.55)	2.20 (0.57)

Race (% white)	93.47 (24.70)	78.5 (15.80)	75.1 (22.90)

Voter participation	-	60.73 (8.55)	58.85 (9.88)

Violent crimes per 100,000 people	-	1153 (714)	1070 (837)

a. Individual data as reported on survey; community-level by reported corresponding zip code and national means [37,42,43].

b. All values for each category at all levels are different (p ≤ 0.025).

c. Education score corresponds to the five education categories described in the methods section; details shown in manuscript 2.

d. Income score corresponds to the five income categories described in the methods section; details shown in manuscript 2.

### Factors related to program implementation: logistic regression analysis

To examine the impact of these measures on program implementation, a logistic regression model was estimated. The logistic equation presented in Tables 7 was specified as: *implementation = educational attainment + program-related self-efficacy + fitness certification/credential + support-focused leadership style + previous strength training experience + age*. These data revealed that leaders who have a fitness credential are approximately twice as likely [OR = 2.3, 95% CI = 1.3–3.9, p = 0.003] to implement the program, and that support-focused leadership style and greater levels of program-related self-efficacy (as measured by the scores previously described) increase the likelihood of implementation (p = 0.006 and p = 0.002, respectively). Additionally, higher educational attainment was negatively associated with program implementation (p = 0.002). Results are shown in Table 7.

**Table 7:** Logistic Regression Model: Factors Related to Program Implementation^a^

**Variables**	**Odd Ratio**	**95% CI**		**p-value**
Educational attainment^b^	0.575	0.408	0.810	0.002

Program-related self-efficacy^c^	2.853	1.483	5.488	0.002

Fitness certification/credential^b^	2.265	1.319	3.891	0.003

Support-focused leadership style^b^	1.305	1.078	1.580	0.006

Previous strength training experience^d^	0.758	0.459	1.251	0.278

Age (years)^b^	1.001	0.979	1.023	0.917

Constant	0.568			0.663

^a ^sample size: n = 314

^b^measured as shown in table 1

^c^measured as shown in table 3

^d^measured as shown in table 2

It is important to note here that the construction of alternative models informed by the univariate results occurred during the analyses of these data. The process involved a phased approach testing for collinearity among variables and using step-wise logistic regression. The variables were first tested for collinearity within their respective categories (socioeconomic, professional, program-related personal/behavior, leadership). For example, within the socioeconomic and professional category, education and income were highly correlated and thus could not be included in any regression models together. Using separate step-wise logistic regression tests, educational attainment remained in the model while income did not; thus, education was chosen over income for inclusion. This also occurred with three variables in the leadership category. Program-related self-efficacy, leader self-identification, and leadership comfort were all correlated, and were subsequently tested in models separately; program-related self-efficacy was chosen for inclusion based upon the model's Cox & Snell R-squared value. The final phase involved testing the remaining variables for potential collinearity followed by step-wise logistic regression. The final model presented (Table 7) is significant (p < 0.001) with a -2 log likelihood of 379.5 and a Cox & Snell R-squared value of 0.125, suggesting that this model may explain approximately 12.5% of the variability in implementation status.

## Discussion

The mission of public health is to prevent disease and promote health in the greater population through the ongoing collection of health-related data; providing sound health information, resources, and recommendations; and supporting the implementation and dissemination of public health initiatives and programming. While we understand the importance of physical activity participation and have data to support the feasibility and benefits of community-based programming [6,12,29,45-52], there is limited evidence related to optimizing implementation rates by leaders. This study sought to describe the characteristics of program leaders from a nationally disseminated program, and to identify and understand factors that help community-based leaders apply what they learn through curricula and trainings to successfully implement programs.

Of approximately forty distinct factors examined – including a range of socioeconomic, professional, personal/behavioral, leadership, and community variables – these data revealed that fitness credentials, support-focused leadership style, and greater levels of program-related self-efficacy were positively associated with program implementation, while higher levels of educational attainment were negatively associated with implementation in a logistic regression analysis. Additionally, in chi square group-level comparisons, physical activity level, perceived support, comfort leading groups, and leader self-identification were higher among implementers compared to non-implementers.

The social ecological model provided a valuable framework to categorize variables related to leader implementation. These findings demonstrated that both individual and interpersonal levels of influence were important. Fitness credentials/certification is an individual leadership characteristic specific to the implementation of this program. For example, leaders in this sample have a variety of professional expertise – from physical and occupation therapy to nursing, dietetics, and chiropractics [6]. In the SWP, fitness credentials are relevant to the program, yet they may not be for other health-promotion/community-based programs. However, it may be that, in general, program-specific training or experience plays an important role in leader confidence as well as competence around the planning, organization, and administration, and/or in execution of the implementing the program and sustaining it.

The self-efficacy score was derived from three questions in which leaders rated their confidence to overcome social, logistical, and physical challenges related to program implementation. Program-related self-efficacy was significantly higher among implementers, which is consistent with previous findings [16,31,53], although scores were quite high among non-implementers as well. Prospective studies would help clarify whether the act of implementation itself increased implementers' self-efficacy scores.

The leadership assessment was asked separately from programmatic questions, and this category of questions was asked in a general context. Respondents were asked to identify potential leadership strengths and weaknesses of their own leadership characteristics in terms of organization, communications, conflict resolution, and providing support, which are commonly examined factors in determining leadership style [17,22,27,40]. In this study, individuals whose leadership style focused on providing support were positively associated with implementation in the logistic regression analysis, while none of the other three categories of leadership were association with implementation. As leaders trained in a community-based health program, support-focused leadership style was a likely characteristic [31]. Future studies might examine similar categories among implementers of other health programs to determine if support-focused leadership remains an important factor. If so, it would be beneficial to include strategies for improving support-focused leadership during trainings, and to consider this leadership characteristic in selection when necessary.

In this study, educational attainment was inversely associated with program implementation. This was an interesting finding and counterintuitive to other findings [3]. However, this is a highly educated cohort, with greater than 82% of individuals having a bachelors degree or higher. In some cases, more than one individual from the same organization was trained. It could be that those in the masters or higher education level category were at the director or administrator level in the organization and therefore their job responsibilities were to oversee programs but not to actually implement them. While not "implementers" themselves, these individuals may have provided mentorship and resource support to leaders who did implement the program, thus acting as critical program advocates who played an essential role in the pathway to implementation. Qualitative data collected in a related study may provide clarification in this area (publication forthcoming).

The primary limitation of this study is the convenience sample and cross-sectional design. This design and sampling structure dictate that all findings are associations in which causality cannot be inferred or implied, and that results cannot be extrapolated to other populations or programs. In future survey research of a similar nature, it would be optimal to administer the survey prior to and following a potential implementation timeframe. Specifically with regard to the sample: they were predominantly women who were mostly white and of relatively high socioeconomic status (SES). Despite this, there is a range within SES. Additionally, the national representation of leaders with varying personal and educational degrees is beneficial for understanding common factors across professions, geographical regions, and urban/rural locations. An additional limitation of this study is response bias. Response bias is a consideration for survey research, although the response rate was 57%. It is helpful that response rate does not appear biased by implementation, as it is similar among implementers and non-implementers.

Although these data provide valuable insights into initial program implementation, there are a number of questions related to program adoption and sustained delivery (i.e. utilization of the RE-AIM Framework) that would provide a fruitful area of further investigation [54,55]. The strengths of these data are that multiple factors from a broad range of categories were examined in the context of a nationally disseminated community-based exercise program. The data offer a comprehensive analysis at the leader level, rather than at the participant outcome-related level, which is perhaps where participant exposure and program success begins.

## Conclusion

Studies on other social change issues have identified a number of factors that are associated with sustained change. These include policy changes, strategic collaborations, and effective leadership at the community level. Without leadership to initiate, motivate, and sustain physical activity programs, the causal pathway to behavior change among community members is interrupted, minimizing the efficacy of programs and reducing the likelihood of community and individual change [4,32,56,57].

Implementation is an important step to increasing access to community programs, and ultimately, to increasing participation. Thus, optimizing implementation rates through leader recruitment and training are viable strategies to achieve that goal. These findings may also help administrators to improve criteria for leader selection, as good leaders are difficult to recruit and maintain.

This study's theoretical model offers a contextual framework of factors related to leader implementation, and contributes knowledge to advance research related to future community-based programs. Utilizing lessons learned from this study, future research – particularly prospective studies – examining a similar social ecological framework that includes personal/behavioral, socioeconomic, professional, leadership, and community factors in other programs and in other populations are clearly warranted. Studies including men and individuals with greater socioeconomic and racial diversity (e.g. low income and non-white populations) would be beneficial to the literature and for applications in a variety of public health programs and settings.

## Competing interests

Authors RA Seguin, R Palombo, CD Economos, R Hyatt and J Kuder declare no competing financial or non-financial interests. Author ME Nelson declares that she is author of eight trade books in the *Strong Women *series. In addition, she is a shareholder in LLuminari, Inc, a women's health media company.

## Authors' contributions

Authors RAS, RP, CDE, RH, and MEN contributed to the concept, design, data analysis, and data interpretation; manuscript development and revisions; and final manuscript approval. JK contributed to data analysis and interpretation; manuscript revisions; and final manuscript approval.
